# The Protective Effects of Water-Soluble Alginic Acid on the N-Terminal of Thymopentin

**DOI:** 10.3390/molecules28186445

**Published:** 2023-09-05

**Authors:** Haiyu Ji, Yuting Fan, Xiaoji Gao, Youshun Gong, Keyao Dai, Zhenhua Wang, Bo Xu, Juan Yu

**Affiliations:** 1 Center for Mitochondria and Healthy Aging, College of Life Sciences, Yantai University, Yantai 264005, China; haiyu11456@163.com (H.J.); fyting7766@163.com (Y.F.); xiaoji0405@163.com (X.G.); gongys8991@163.com (Y.G.); skywzh@ytu.edu.cn (Z.W.); xubo168@sina.com (B.X.); 2 College of Food Science and Engineering, Tianjin University of Science and Technology, Tianjin 300457, China; dai13389086120@163.com

**Keywords:** water-soluble alginic acid, thymopentin, immune potentiation

## Abstract

Thymopentin (TP5) has exhibited strong antitumor and immunomodulatory effects in vivo. However, the polypeptide is rapidly degraded by protease and aminopeptidase within a minute at the N-terminal of TP5, resulting in severe limitations for further practical applications. In this study, the protective effects of water-soluble alginic acid (WSAA) on the N-terminal of TP5 were investigated by establishing an H22 tumor-bearing mice model and determining thymus, spleen, and liver indices, immune cells activities, TNF-α, IFN-γ, IL-2, and IL-4 levels, and cell cycle distributions. The results demonstrated that WSAA+TP5 groups exhibited the obvious advantages of the individual treatments and showed superior antitumor effects on H22 tumor-bearing mice by effectively protecting the immune organs, activating CD4^+^ T cells and CD19^+^ B cells, and promoting immune-related cytokines secretions, finally resulting in the high apoptotic rates of H22 cells through arresting them in S phase. These data suggest that WSAA could effectively protect the N-terminal of TP5, thereby improving its antitumor and immunoregulatory activities, which indicates that WSAA has the potential to be applied in patients bearing cancer or immune deficiency diseases as a novel immunologic adjuvant.

## 1. Introduction

Alginic acid (AA) is a natural polysaccharide aldehyde acid which is composed of β-d-mannuronic acid (M) and α-l-guronuronic acid (G) formed by 1, 4-linkage with no regular repeating units and is commonly found in the cell walls of brown algae [1,2]. AA is known as one of the most famous polysaccharides due to its characteristics, including being biocompatible, biodegradable, non-toxic, and low-cost [3,4]. Recent research has proved that AA could exhibit immunomodulatory, antioxidant, and anti-inflammatory effects [5,6,7]. However, the naturally extracted AA has always presented larger molecular weights and lower water solubility, which has severely limited its practical application [8]. Furthermore, sodium alginate has mainly existed in the form of seaweeds, which lost the characteristics of acidic polysaccharides [9]. Therefore, in this paper, a water-soluble alginic acid (WSAA) presenting superior water solubility was prepared which had the potential to exhibit better biological activities.

The bioactive pentapeptide thymopentin (TP5), with the chemical formula Arg-Lys-Asp-Val-Tyr and a molecular weight of 679.77, exhibits excellent water solubility and possesses notable biological properties, including its ability to facilitate CD4^+^ and CD8^+^ subsets differentiation and maturation [10,11,12], which have been used for treating immunodeficiency diseases including cancer, etc. [13,14]. Furthermore, TP5 has demonstrated robust immunomodulatory activity by stimulating other immune cells [15]. Nevertheless, the half-life of TP5 in vivo after intramuscular or percutaneous injection is ≤30 s, which has limited its application severely [16,17]. Thus, the development of novel adjuvants is imperative in order to improve the efficacy of TP5.

Hepatocellular carcinoma, which is primarily induced through chronic viral infections [18], is the leading cause of death in mainly Asian/African countries and its morbidity ranks fifth in the world [19,20]. Chemotherapy has been the commonly employed therapeutic strategy for various cancer treatments, while the development of tumor cell resistance to chemotherapeutics can significantly decrease the efficacy of treatment [21,22]. As reported, cancer immunotherapy could effectively eliminate cancer cells with non-toxic side effects [23,24]. Therefore, the development of antitumor immunomodulators and the enhancement of immune activity have attracted the attention of a lot of scholars aiming to inhibit tumor growth and relieve the suffering of patients [25].

The current study aimed to investigate the protective effects of WSAA on the N-terminal of TP5 by preparing a complex of WSAA and TP5, evaluating its antitumor effects through the construction of a solid tumor mice model. Our findings hold promise for developing an exceptional immunopotentiator for clinical therapeutics targeting TP5 in cancer patients. In recent years, there has been a growing interest for exploring novel therapeutic approaches for cancer treatments. This study aimed to develop the potential benefits offered by WSAA when combined with TP5, specifically focusing on its protective effects on the N-terminal region. By creating a complex between WSAA and TP5, researchers were able to evaluate their collective impact on inhibiting tumor growth.

To assess the effectiveness of this combination therapy (WSAA–TP5 complex), an H22 solid tumor mice model was constructed. The results provide valuable insights into how this immunopotentiator can be utilized as a potential therapeutic option for cancer patients, and the findings have significant implications for future clinical applications targeting TP5 in cancer patients. Further research is warranted to fully understand the mechanisms underlying these protective effects and optimize dosage regimens.

## 2. Results

### 2.1. Preliminary Structural Analysis of WSAA

**Figure 1:**
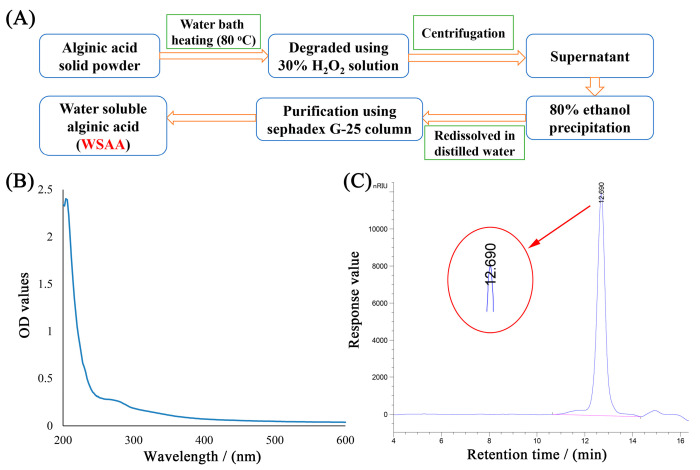
Preparation technology (**A**), UV spectrum (**B**), and HPGPC profile (**C**) of WSAA.

The UV/HPGPC methods were utilized to conduct a preliminary structural analysis of WSAA, and the results are presented in Figure 1B,C. The absence of absorption peaks at 260 nm or 280 nm in the UV spectrum of WSAA shown in Figure 1B indicates that there was little nucleic acid or protein present in the prepared WSAA, suggesting that the WSAA presented high purity, which was essential for the further investigations.

Furthermore, Figure 1C demonstrated that the average molecular weight of WSAA was 6.48 × 10^3^ Da. This information provides insight into the physical properties of WSAA and can be used to optimize its performance for various applications. Overall, these findings are crucial steps towards understanding the structure and characteristics of WSAA.

### 2.2. Schematic Diagrams of WSAA–TP5 Complex

**Figure 2:**
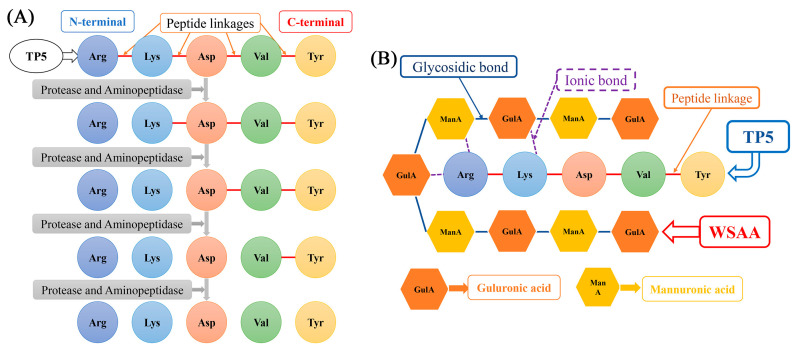
Schematic diagrams of TP5 degradation (**A**) and WSAA protecting TP5 (**B**).

The half-life of TP5 in vivo after intramuscular or percutaneous injection is generally ≤30 s. This short half-life can be attributed to the rapid degradation of TP5 by protease and aminopeptidase enzymes from the N-terminal region. Such enzymatic degradation severely limits the practical application of TP5 [26]. To address this limitation, Figure 2A presents a diagram illustrating the degradation process. However, an alternative approach has been explored to enhance the stability and bioactivity of TP5. As depicted in Figure 2B, it could be observed that when WSAA (water-soluble alginic acid) was combined with the N-terminal of TP5 through electrovalent bonds, they could provide protection against enzymatic degradation. This interaction between WSAA and TP5 not only prevents degradation but also contributes to stronger bioactivities exhibited by TP5. By shielding the vulnerable N-terminal region from proteases and aminopeptidases, WSAA effectively preserves the structural integrity and functional properties of TP5. These findings suggest that modifying or incorporating WSAA into peptide-based drugs like TP5 could potentially overcome the limitations related to rapid enzymatic degradation.

### 2.3. Organs Indices and Tumors Inhibitory Rates

**Figure 3:**
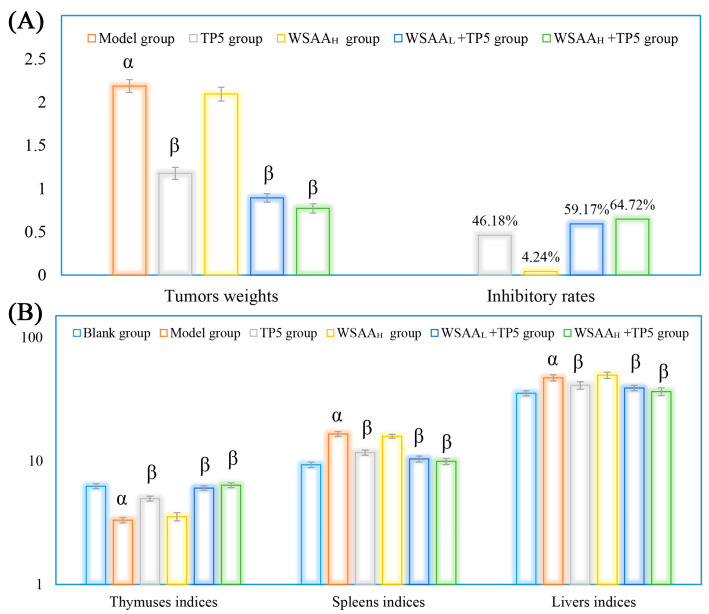
Tumor inhibitory rates (**A**) and organs indices (**B**) of mice in these groups. Note: α, *p* < 0.05 compared with blank group; β, *p* < 0.05 compared with model group.

Figure 3A shows the H22 tumors’ weights and inhibitory rates in mice. The mice in the model group only received saline solution (during 1~28 d) and H22 cells (on 14 d). As presented, the average tumor’s weight in mice in the model group increased to 2.18 g rapidly, while TP5 presented strong inhibitory effects on solid tumor growth (inhibitory rates of 46.18%) by enhancing antitumor immunity, and individual WSAA treatment showed little antitumor effect in vivo. The WSAA_L_/WSAA_H_ groups received hypodermic injections of WSAA at dosages of 5 mg/kg and 10 mg/kg, respectively. When combined with TP5 treatment, WSAA_L_ and WSAA_H_ showed lower solid tumor weights and higher inhibitory rates in mice than individual TP5 injection, and the WSAA_H_+TP5 group exhibited an inhibitory rate of 64.72%.

As shown in Figure 3B, the H22 tumor cells’ proliferation in the model group induced the atrophy of thymuses and the tumefactions of spleens/livers, resulting in the dysfunction of these organs compared to those of the blank group. After TP5 treatment, compared to the model group, the thymus indices were significantly increased while the spleens/livers indices were remarkably decreased, suggesting that TP5 could effectively protect body organs in H22 tumor-bearing mice. Additionally, in combination with different concentrations of WSAA, the organ conditions of mice were further improved than the individual TP5 group’s, indicating remarkable immune potentiation by the WSAA–TP5 complex.

### 2.4. Routine Analysis of Blood

The results of blood routine examinations for H22 tumor-bearing mice in these groups following various drug treatments are presented in Table 1.

The study found that the H22 tumor-bearing mice in the model group had increased levels of granulocytes and platelets, while the lymphocytes and hemoglobin levels were reduced, suggesting that cancer cells could inhibit lymphocyte activity and lead to anemia and inflammation. However, treatment with TP5 showed effective enhancements in lymphocytic immunity and relieved adverse effects. Interestingly, when combined with WSAA, the complex-stimulated H22 tumor-bearing mice showed remarkably enhanced antitumor immunity in a dose-dependent manner. These findings suggest that combination therapy may be more effective than individual treatments for enhancing immune function in cancer patients.

### 2.5. Lymphocyte Subsets Detection

**Figure 4:**
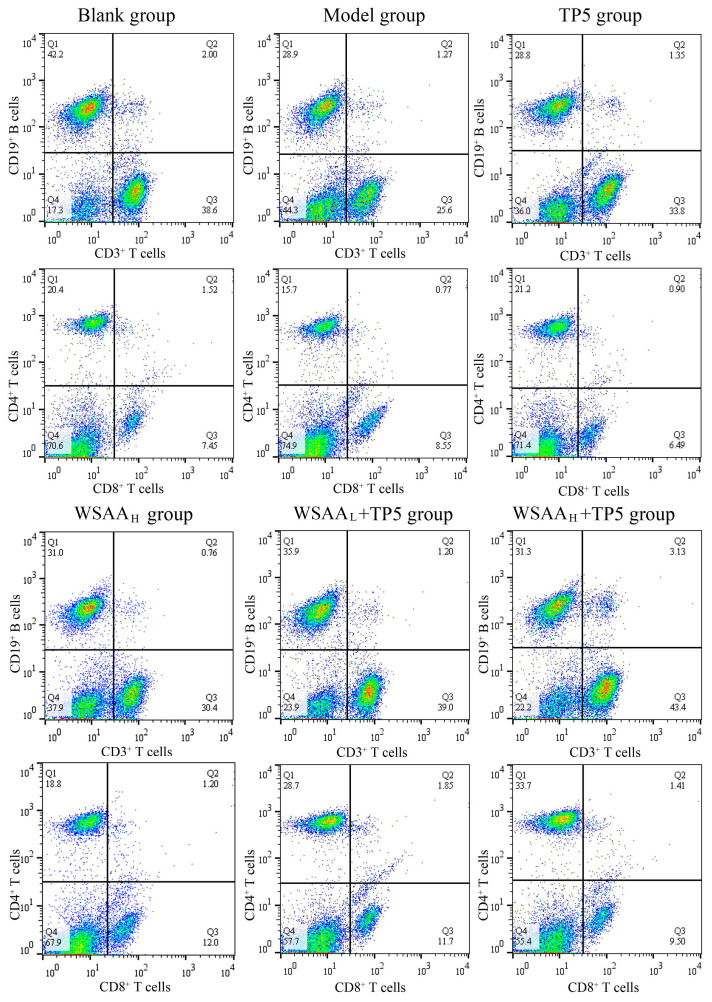
Distributions and proportions of CD3^+^ T and CD19^+^ B lymphocytes and CD4^+^ T and CD8^+^ T cells in peripheral bloods.

The lymphocyte subset distributions and proportions in the bloods of each group were determined and the results are shown in Figure 4. As presented, the percentages of CD19^+^ B cells, CD3^+^ T cells, and CD3^+^CD4^+^ T cells in the model group were obviously decreased compared with that in the blank group, while CD3^+^CD8^+^ T cell proportions showed inconspicuous differences, which indicates that the CD19^+^ B cells’ and CD3^+^CD4^+^ T cells’ activities were suppressed by the H22 solid tumors. After individual TP5 treatment, the proportions of CD3^+^ T cells and CD3^+^CD4^+^ T cells were obviously increased compared with that of the model group. Additionally, the combination of TP5 and WSAA displayed stronger immunoregulatory activity compared with individual TP5 treatment, and CD19^+^ B cells’, CD3^+^ T cells’, and CD4^+^ T cells’ proportions were all remarkably improved, indicating the enhanced antitumor immune responses of CD19^+^ B cells and CD4^+^ T cells in vivo.

### 2.6. Lymphocytes Proliferation Activities and Antibodies Levels

**Figure 5:**
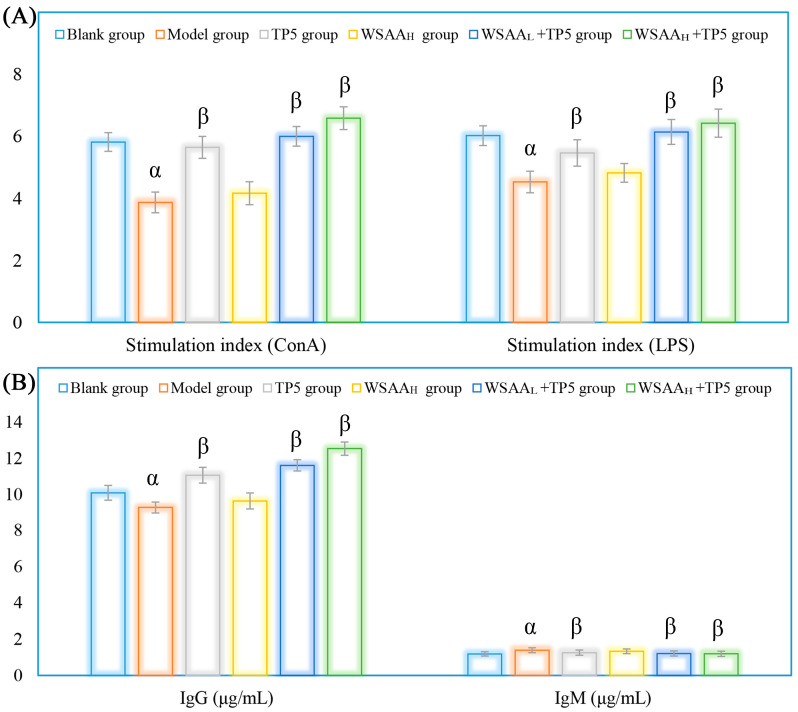
Proliferative activities of splenic T and B lymphocytes (**A**) and antibodies expressions (**B**) in sera of mice in these groups. Note: α, *p* < 0.05 compared with blank group; β, *p* < 0.05 compared with model group.

As depicted in Figure 5A, the uncontrolled proliferation of H22 tumors in the model group resulted in suppressed splenic T/B lymphocyte proliferation compared to that of the blank group stimulated by Con A/LPS. However, after treatment with TP5, there were significant improvements in lymphocyte proliferation activities compared to that of the model group. After being treated with TP5 and WSAA, the stimulation indices of splenic lymphocytes were all remarkably increased compared with the separate treatment group, indicating their significant synergism in H22 tumor-bearing mice. As depicted in Figure 5B, the levels of IgG/IgM in the sera of model groups were significantly decreased/increased compared to those of the blank group, indicating their distinct indicative functions (IgG reflecting anti-tumor ability and IgM reflecting H22 tumor cell amounts). However, after treatment with TP5 combined with WSAA, the levels of IgG/IgM in the sera of the model groups were significantly increased/decreased compared to those of the model group, suggesting that WSAA–TP5 treatments could effectively enhance antitumor capacities in H22 tumor-bearing mice while suppressing cancer cell quantities.

### 2.7. Macrophages and NK Cells Activities

**Figure 6:**
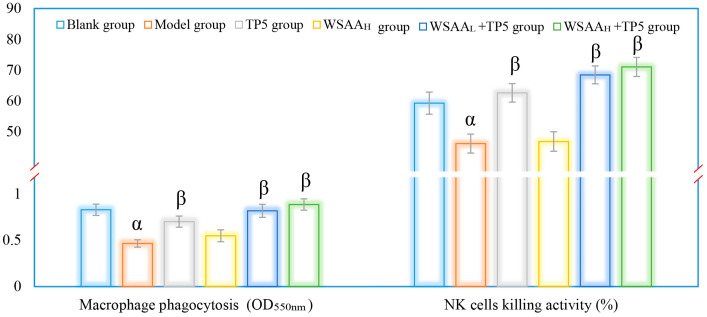
The activities of peritoneal macrophages and splenic NK cells in mice of these groups. Note: α, *p* < 0.05 compared with blank group; β, *p* < 0.05 compared with model group.

The activities of mice macrophages and NK cells were assessed in this study to evaluate the effects of TP5 and WSAA on immune responses [27]. The results, as depicted in Figure 6, showed similar trends in these groups. It was observed that the model group exhibited a significant decrease in macrophage phagocytosis and NK cell killing activities compared to the blank group; the decline could be attributed to the uncontrolled multiplication of H22 solid tumor cells. However, when treated with TP5 alone, there was a notable improvement in the immunological functions of both macrophages and NK cells. The phagocytic ability of macrophages increased significantly, while the cytotoxicity of the NK cells also improved compared to the model group, indicating that TP5 treatment enhanced immune responses by boosting these two types of immune cells. Interestingly, when comparing the TP5 group with the combination therapy using both TP5 and WSAA, it was found that their immunoregulatory activities in H22 tumor-bearing mice were even stronger, suggesting that WSAA had additional positive impacts on enhancing immune responses when combined with TP5 stimulation for mice bearing tumors. These results demonstrate that both TP5 and WSAA have significant immunological enhancement effects on mice with H22 solid tumors.

### 2.8. Cytokines Levels in Sera

**Figure 7:**
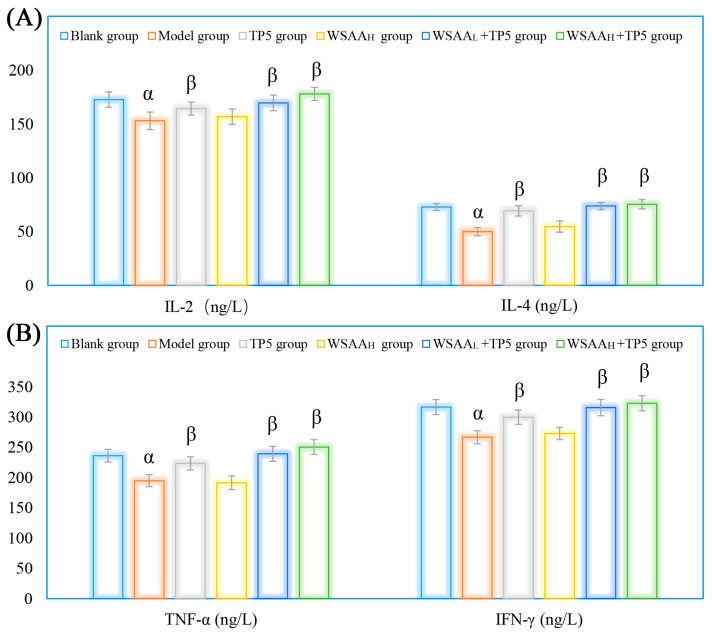
Expression levels of IL-2 and IL-4 (**A**) and TNF-α and IFN-γ (**B**) in mice sera. Note: α, *p* < 0.05 compared with blank group; β, *p* < 0.05 compared with model group.

As shown in Figure 7A,B, the results indicate that four types of cytokines exhibited similar relationships with the eliminative capacities of solid tumor cells in vivo. The expression levels of IL-2, IL-4, TNF-α, and IFN-γ in the sera of the model group were significantly decreased compared to the blank group. This decrease in cytokine levels could potentially promote H22 cells proliferation. However, TP5 treatment was found to effectively increase these immune-related cytokine levels in mice, thereby inhibiting the growth of H22 tumors. Furthermore, when combined with WSAA, it was observed that the levels of these cytokines were further improved compared to those seen with individual treatments. These findings highlight the potential therapeutic benefits of TP5 and its combination with WSAA for cancer treatment. By increasing immune-related cytokine levels, this approach might help suppress tumor growth and enhance overall antitumor responses. Nonetheless, these results could provide valuable insights into potential strategies for improving cancer therapy through the modulation of immune responses [28].

### 2.9. Cell Cycle Determination

**Figure 8:**
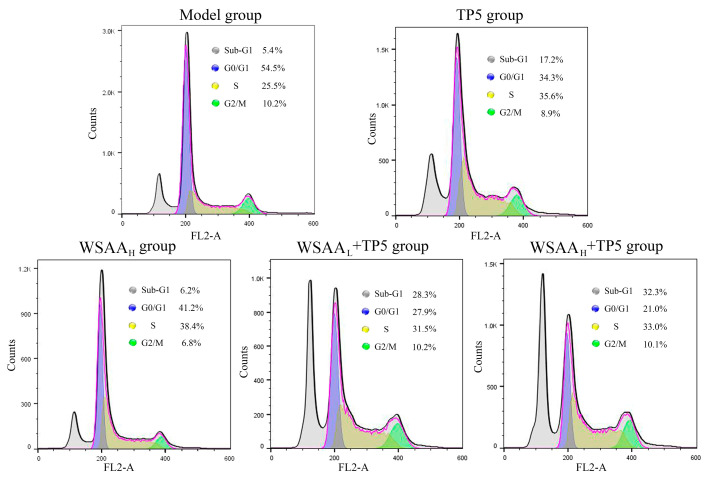
Cell cycle distributions and apoptosis rates of H22 solid tumor cells in mice of these groups.

The cell cycle of mice H22 solid tumor cells was determined, and the proportions of cells in Sub-G1, G0/G1, S, and G2/M phases are displayed in Figure 8. As presented, the apoptotic rate of solid tumor cells in the model group was 5.4%, which might result from the grinding process of solid tumors for single-cell suspension preparation, and the percentages of G0/G1, S, and G2/M phases were 54.5%, 25.5%, and 10.2%, respectively. After individual TP5 treatment, the apoptotic rates of H22 solid tumor cells increased to 17.2%, indicating their immunomodulatory effects. Furthermore, the proportions of H22 cells in S phase obviously increased to 35.6%, which suggests that the apoptosis was induced by S phase retardation. Compared with the TP5 group, the combination of WSAA with low and high dosages could further improve the apoptotic rates of tumor cells to 28.3% and 32.3%, which were also induced by arresting them at the S phase.

## 3. Discussion

AA is composed of D-mannuronic and L-guluronic acid with β-1→4-linkage, and the chain length would be different depending on extraction conditions, which could also significantly affect its physicochemical properties and biological functions [29]. TP5 is an immunocompetent polypeptide with no side effects and has been widely applied to improve immunological indices in hosts bearing immune deficiency diseases [30]. The C-terminus and N-terminus of TP5 are characterized by carboxyl groups and amino groups, respectively, while the degradation of TP5 primarily occurs at the N-terminus through protease and aminopeptidase activities. Interestingly, the use of cationic compounds to protect the C-terminus does not impede the degradation rate of thymopentin, as supported by our previous findings that low-molecular-weight chitosan actually accelerates its in vivo degradation, consequently diminishing its immune-regulatory bioactivity [31]. In this paper, WSAA was prepared using H_2_O_2_ degradation, and the model of mice suffering H22 solid tumors was constructed, and the protective effects of WSAA on the N-terminal of TP5 were evaluated through immunological indicator determination.

The thymus and spleen, being vital immune organs in the body, play a crucial role in safeguarding against infections and cancers by activating diverse populations of immune cells [32]. The liver is an organ that primarily functions in metabolism and hematopoiesis [33]. As reported, anemia, infection, and suppressed immune cells activities are common complications in cancer-bearing patients [34,35]. In this study, the individual TP5 treatment exhibited strong antitumor activity and significantly reduced the solid-tumors-induced side effects as expected [36]. Additionally, the combination groups displayed higher inhibitory effects in mice bearing H22 solid tumors, indicating that the WSAA presented strong immune potentiation in TP5-stimulated mice.

Macrophages are mainly composed of pro-inflammatory (M1) and anti-inflammatory (M2) cells, which can treat various microenvironment signals [37]. NK cells demonstrate important antitumor responses through their direct killing ability and releasing inhibitory cytokines [38]. As reported, CD4^+^ T cells are the primary subsets of T cells and can exert indirect inhibitory effects on tumor cells, while the CD8^+^ T cells are the main effector cells [39]. Moreover, B cells mediate humoral immunity by secreting a variety of antibodies including IgG and IgM, which play important roles in the antitumor effects of the body [40]. In this study, TP5 effectively inhibited the growth of solid tumors in mice and presented strong immunological enhancements on macrophages, NK cells, and CD4^+^ T cells. Furthermore, WSAA combined groups also showed higher B cells proportions and proliferation abilities, as well as IgG antibodies levels, and finally resulted in higher inhibitory effects on tumor cells. Based on the evaluation of tumor development in different groups of mice, it can be inferred that IgM antibodies did not exhibit any distinct inhibitory effects on tumor cells, while IgG antibodies played a more significant role in effectively eliminating tumor cells [41,42].

Cytokines are acknowledged as signaling molecules that play vital roles in intercellular communication within the immune system and various other biological processes. They can be proteins or peptides secreted by different cell types, including immune cells, in response to diverse stimuli such as infections, injuries, or immune responses. Examples of cytokines encompass interleukins (IL-2, IL-6, IL-8, IL-12), TNF-α, and IFNs [43,44,45]. TNF-α participates in the destruction of tumor cell structure and the induction of cell apoptosis and may serve as a co-stimulatory factor for mitogen-activated normal B cells [46]. IFN-γ is mainly produced by T cells and NK cells and plays a crucial role in antitumor immunity by activating lymphocytes, macrophages, and NK cells [47,48]. IL-2 could promote the tumor-specific NK cells and T cells activities, and IL-4 could regulate immune responses [49,50]. In the present study, TP5 significantly improved the sera cytokine levels of mice bearing H22 solid tumors as expected, thereby inhibiting solid tumor growth by arresting the S phase. Furthermore, the combined treatments of TP5 and WSAA showed stronger immunopotentiation in the cytokine expressions, suggesting that the complex could effectively enhance antitumor immune responses even compared with the individual TP5 treatment group. However, the apoptosis mechanisms of tumor cells under immune system attack in vivo still need to be further researched.

## 4. Materials and Methods

### 4.1. Materials and Reagents

The TP5 was chemically synthesized by Beijing Protein Innovation Co., Ltd. (Beijing, China), and the purity surpassed 98%. Anti-mouse monoclonal antibodies of CD3-FITC, CD19-PE, CD8-FITC, and CD4-PE were provided by the BioLegend company (San Diego, CA, USA); the alginic acid (AA), Mouse Spleen NK Cells Isolation Kit, and MTT Cell Proliferation and Cytotoxicity Assay Kit were purchased from Beijing Solarbio Science & Technology Co., Ltd. (Beijing, China); the Immunoglobulin G Assay Kit, Immunoglobulin M Assay Kit, Tumor Necrosis Factor-α (TNF-α) Assay Kit, Interferon-γ (IFN-γ) Assay Kit, Interleukin-2 (IL-2) Assay Kit, and Interleukin-4 (IL-4) Assay Kit were provided by the Nanjing Jiancheng Bioengineering Institute (Nanjing, Jiangsu province, China). However, the other reagents used in the present paper were of analytical grade.

### 4.2. Preparation of WSAA

The WSAA was prepared by following the procedure shown in Figure 1A. In brief, the alginic acid powder was immersed in a 30% H_2_O_2_ solution in an 80 °C water bath for 10 h; then the supernatant was gathered using the centrifugation method and precipitated by adding ethanol 4 times its volume. Subsequently, the sediment was redissolved in distilled water and isolated with the Sephadex G-25 column to acquire WSAA. Finally, the purity of the WSAA was initially determined with the ultraviolet spectrum of the UV-2500PC UV-Vis spectrophotometer scanning from 200 nm to 800 nm (Shimadzu, Kyoto, Japan).

### 4.3. Molecular Weight Determination of WSAA

The molecular weight of the prepared WSAA was determined through the employment of high-performance gel permeation chromatography (HPGPC, Agilent-1200 series, Santa Clara, CA, USA) with a TSK-gel G4000PW×L column (7.8 mm × 300 mm) [51]. The operation parameters were set as follows: sample loading volume of 20 μL, eluent flow rate of 0.8 mL/min, column temperature of 30 °C, and Differential Refractive Index Detector (RID) temperature of 35 °C. Additionally, T-series dextrans including T110 (110 kDa), T70 (70 kDa), T40 (40 kDa), T10 (10 kDa), and T3 (3 kDa) were used as standards.

### 4.4. Design of Animal Experiment Program

Sixty female BALB/c mice (18–22 g) were provided by SPF (Beijing) Biotechnology Co., Ltd. (Beijing, China), and raised under a relative humidity of 45–55% and controllable temperatures of 20–25 °C with a 12 h light/12 h dark cycle. The mice were bred in an environment with controlled temperature (20–25 °C), relative humidity (45–55%), and a 12-h light/dark cycle. They were randomly divided into six groups, each consisting of ten mice: Blank group, Model group, TP5 group, WSAA_H_ group, WSAA_L_+TP5 group, and WSAA_H_+TP5 group. The blank and model groups received a saline solution, while the TP5-related groups were given subcutaneous injections of TP5 at a dosage of 1 mg/kg once daily. The WSAA_L_/WSAA_H_ groups received hypodermic injections of WSAA at dosages of 5 mg/kg and 10 mg/kg, respectively. After 14 days of treatment, H22 cells with a concentration of 2 × 10^6^ cells/mouse were injected into the right axilla of all mice except for the blank group followed by another fourteen days’ treatment period.

### 4.5. Analysis of Physiological Indices

Upon the trial’s conclusion, measurements were taken of the mice’s body weight, thymus weight, spleen weight, liver weight, and tumor weight. The following formula was employed to determine the inhibitory rates: Inhibitory rates (%) = (M_1_ – M_2_)/M_1_ × 100, where M_1_ and M_2_ represent the mean tumor weights of the model group and treatment groups. These measurements were subsequently utilized to calculate organ indices based on their respective body weights.

### 4.6. Blood Routine Examination

Blood routine examination is a widely utilized method for assessing the quantity and quality of white blood cells, their subsets, red blood cells, and platelets. Following the immediate addition of EDTA-K2 to obtain fresh mouse blood samples and prevent coagulation, an automated blood cell analyzer was employed to examine them in accordance with mice patterns.

### 4.7. Lymphocytes Subsets Determination

The PE-CD19, FITC-CD3, PE-CD4, and FITC-CD8 antibodies were applied to stain white blood cells after the removal of red blood cells; fluorescence-labeled subsets were gathered and determined [52] and then analyzed using the FlowJo software (10.8.1).

### 4.8. Immune Cells Activities and IgG/IgM Expressions

The splenic lymphocyte proliferation capacities, peritoneal macrophage phagocytic capacities, and NK cells’ killing activities were determined according to previously reported methods [31,53].

Additionally, the expression levels of IgG and IgM antibodies in sera of mice were determined using the corresponding ELISA kits following the instructions, and then the results were further calculated and analyzed.

### 4.9. Cytokines Levels Evaluation and Cell Cycle Detection

The IL-2, IL-4, TNF-α, and IFN-γ levels were determined and calculated according to the kits’ instructions. Additionally, the solid tumor cells of mice were prepared by grinding and filtering through a 300-mesh cell strainer, and the cell cycle distributions were evaluated using the DNA Content Quantitation Kit (Solarbio Life Science, Beijing, China).

### 4.10. Statistical Analysis

All experimental data were presented as the mean ± standard deviation (S.D.), and statistical analysis was performed using the SPSS 20.0; ANOVA and Duncan’s multiple range test (*p* < 0.05) were used for other analyses of significant differences.

## 5. Conclusions

In conclusion, the water-soluble alginic acid (WSAA) with low molecular weight (6.48 × 10^3^ Da) was isolated and purified via H_2_O_2_ degradation; the WSAA–TP5 complex showed superior antitumor effects on H22 tumor-bearing mice by effectively protecting immune organs, activating immune cells, and promoting immune-related cytokines expressions compared with the individual TP5 treatment group, finally resulting in the apoptosis of H22 cells by arresting them in S phase, indicating that WSAA could obviously protect the N-terminal of TP5 and thereby improve its antitumor and immunoregulatory activities. Therefore, WSAA could be used in cancer-bearing patients or other related immunocompromised diseases as a novel immunologic adjuvant.

## Figures and Tables

**Table 1:** The blood routine examination results of H22 tumor-bearing mice in each group.

Items	Units	Blank Group	Model Group	TP5 Group	WSAA_H_ Group	WSAA_L_+TP5 Group	WSAA_H_+TP5 Group
Leukocyte	10^9^/L	5.63 ± 1.01	8.97 ± 1.52 ^α^	7.60 ± 1.36 ^β^	9.15 ± 1.46	6.53 ± 1.25 ^β^	6.30 ± 1.41 ^β^
Lymphocyte proportion	%	72.29 ± 4.62	53.73 ± 5.26 ^α^	64.74 ± 5.15 ^β^	52.79 ± 4.63	69.22 ± 4.85 ^β^	74.44 ± 6.10 ^β^
Intermediate cell proportion	%	2.49 ± 0.12	1.90 ± 0.13 ^α^	1.84 ± 0.16	1.64 ± 0.18	1.84 ± 0.13	2.54 ± 0.19 ^β^
Granulocyte proportion	%	25.22 ± 1.83	44.37 ± 3.69 ^α^	33.42 ± 3.10 ^β^	45.57 ± 2.96	28.94 ± 2.12 ^β^	23.02 ± 2.05 ^β^
Erythrocyte	10^12^/L	9.56 ± 0.62	7.18 ± 0.46 ^α^	8.24 ± 0.49	8.59 ± 0.45	9.19 ± 0.68 ^β^	9.28 ± 0.67 ^β^
Hemoglobin	g/L	181.63 ± 9.68	158.70 ± 8.79 ^α^	165.79 ± 8.07 ^β^	159.49 ± 8.01	170.41 ± 10.67 ^β^	174.55 ± 10.09 ^β^
Mean corpuscular hemoglobin concentration	g/L	374.78 ± 20.95	351.40 ± 26.63 ^α^	365.80 ± 16.09 ^β^	354.52 ± 19.37	370.11 ± 20.78 ^β^	375.23 ± 23.65 ^β^
Platelet	10^9^/L	353.23 ± 16.98	421.67 ± 21.84 ^α^	388.94 ± 20.59 ^β^	419.53 ± 29.38	376.23 ± 26.49 ^β^	368.97 ± 20.91 ^β^

Note: ^α^, *p* < 0.05 compared with blank group; ^β^, *p* < 0.05 compared with model group.

## Data Availability

Data sharing not applicable.

## Sample Availability

Samples of the compounds are available from the authors.
